# Chronic Lymphocytic Leukemia Causing Gastric Ulcer Perforation: A Case Presentation and Literature Review

**DOI:** 10.7759/cureus.36026

**Published:** 2023-03-11

**Authors:** Ariana R Tagliaferri, Gabriel Melki, Walid Baddoura

**Affiliations:** 1 Internal Medicine, St. Joseph's Regional Medical Center, Paterson, USA; 2 Medicine, St. Joseph's University Medical Center, Paterson, USA; 3 Gastroenterology, St. Joseph's Regional Medical Center, Paterson, USA

**Keywords:** stomach ulcer, non-hodgkin’s lymphoma, leukemia, gastric ulcer perforation, cll

## Abstract

Chronic lymphocytic leukemia (CLL) is a malignancy characterized by the progressive accumulation of lymphocytes in the bone marrow and lymphoid organs. Gastrointestinal manifestations are rare in all types of leukemia. Generally, this occurs during relapsing disease or in acute leukemias; however, recent advancements in treatment have reduced these complications. Most commonly, lesions in the stomach are hemorrhagic, and lesions in the lower gastrointestinal tract present as peritonitis or colitis. Our patient was unique because she had a perforated, rather than bleeding, peptic ulcer caused by infiltrative chronic lymphocytic leukemia after starting ibrutinib. Although this medication can impair wound healing and/or cause bleeding, there are no reports of perforation of existing ulcers. Additionally, chronic lymphocytic leukemia causing perforated peptic ulcer disease (PUD) is rare, and this is, to our knowledge, the first case of this phenomenon.

## Introduction

Chronic lymphocytic leukemia (CLL) is a malignancy characterized by the progressive accumulation of lymphocytes in the bone marrow and lymphoid organs [1, 2]. It was first described in the 1950s and has since become the most common form of leukemia in adults, accounting for approximately 25% of all adult leukemia and 25% of non-Hodgkin’s lymphoma in the western world [1, 2]. Patients are typically diagnosed in their fifth to sixth decade of life and may be asymptomatic or present with non-specific symptoms, such as fatigue or recurrent infections [1, 2]. Clinically, there may be hepatosplenomegaly, leukocytosis, lymphocytosis, anemia, and thrombocytopenia [1].

The diagnosis of CLL is based on the presence of monoclonal B-lymphocytes > 5,000 lymphocytes/uL in the peripheral blood for at least three months and < 55% prolymphocytes and flow cytometry positivity for clusters of differentiation (CD) 5, CD19, CD20, and CD23 [1]. Previously, CLL was treated with steroids, chlorambucil, or fludarabine; however, recent advancements have led to the approval of bruton tyrosine kinase inhibitors, such as ibrutinib, B-cell lymphoma (Bcl-2) antagonists, and anti-CD20 monoclonal antibodies, such as obinutuzumab, for the treatment of initial and/or relapsing CLL [1]. The advancements and progress have continued, and other targeted treatments now include p110 PI3Kδ inhibitors, such as idelalisib, or spleen tyrosine kinase (SYK) inhibitors [1]. 

Rarely has CLL been described with gastrointestinal involvement, with the majority of gastrointestinal malignancies being mantle cell or T-cell lymphomas [3]. Only 25% of all types of leukemia are complicated by gastrointestinal manifestations [2]. Generally, such occurrences are during relapsing disease or acute leukemia; however, the incidence has been decreasing due to advancements in and efficacy of treatment [2]. In the few instances where CLL has been reported in the gastrointestinal tract, the most common lesions are found in the stomach, ileum, or proximal colon and are likely to be hemorrhagic in the upper gastrointestinal tract or peritonitis/colitis in the lower gastrointestinal tract [2]. This is, to our knowledge, the first case of CLL causing a perforation of a peptic ulcer after starting ibrutinib.

## Case presentation

A 72-year-old female with a past medical history of chronic lymphocytic leukemia (CLL), warm autoimmune hemolytic anemia, hypertension, and hyperlipidemia presented to the emergency department (ED) with sharp abdominal pain lasting one day. The pain was located in the epigastric region and radiated to all quadrants. It was associated with nausea and two non-bloody episodes of vomiting. She denied fevers, chills, recent sick contacts, hematochezia, or melena. She was started on ibrutinib for her CLL due to steroid-refractory autoimmune hemolytic anemia a month prior to admission. Approximately three months prior to admission, she was initially diagnosed with autoimmune hemolytic anemia, reported a 50-pound weight loss, and fatigue, and was found to have bilateral inguinal and axillary lymphadenopathy. At that time, she was also noted to have macrocytic anemia (hemoglobin: 5.6 mg/dL, mean corpuscular volume (MCV): 126, hematocrit: 16.5%), thrombocytopenia (133 K/mm^3), elevated lactate dehydrogenase (305 U/L), a reticulocyte count (7.01%), lymphocytosis (white blood cells (WBC) of 21.1 x 10^3/mm3) and spherocytes on a peripheral blood smear. The Coombs test was positive for IgG and C3. A flow cytometry analysis was positive for clonal kappa, CD5(+)/CD23(+) B-cell population (approximately 71%), with CD38 expression detected on B-cells and ZAP-70+, consistent with chronic lymphocytic leukemia.

On arrival, she was afebrile and normotensive (121/79 mm Hg) with a heart rate of 74 beats per minute. All other vital signs were stable. The abdomen was soft, non-distended, and tender to deep palpation of the right lower quadrant. Laboratory studies revealed macrocytic anemia, borderline thrombocytopenia, and leukopenia (Table 1).

**Table 1:** Admission laboratory values based on a complete blood count Shown are admission laboratory values from the complete blood count demonstrating hematological errors consistent with baseline CLL and autoimmune hemolytic anemia.

Laboratory test	Laboratory value	Reference range
White blood cell count	3.0 x10^3/mm3	4.5-11.0 x10^3/mm3
Red blood cell count	3.05	4.0-5.33 x10^6/mm3
Hemoglobin	9.7 mg/dL	12.0- 16.0 mg/dL
Hematocrit	30.8%	36-46%
Mean corpuscular volume	100.8 fL	80-100 fL
Red cell distribution width	15.9 %	0.5-16.5 %
Platelets	159 K/mm^3	140-440 K/mm^3

**Figure 1:**
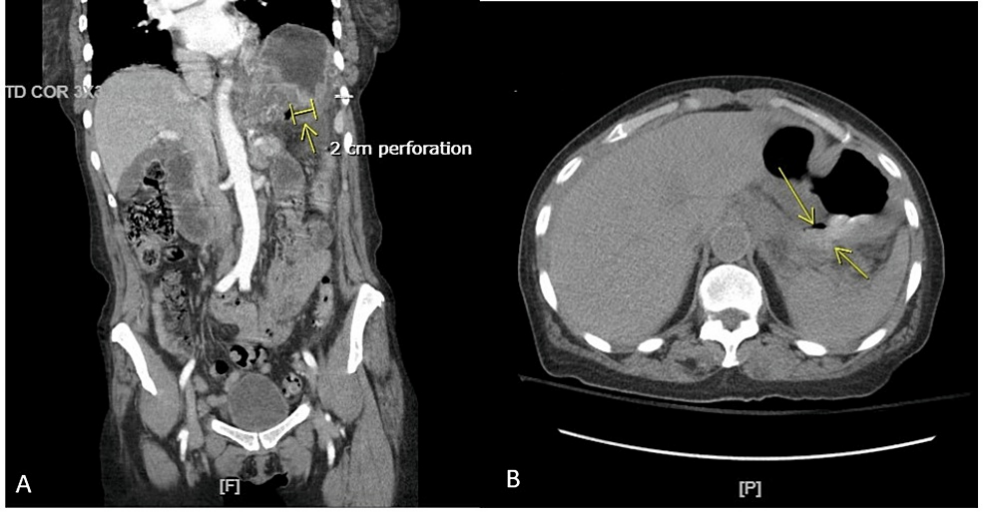
A computerized tomography scan of the abdomen and pelvis showing a gastric ulcer perforation. Figure 1A is a coronal view of the computerized tomography scan of the abdomen and pelvis with intravenous contrast and oral contrast administered prior to examination. The arrow represents an ulcer perforation along the posterior-inferior gastric wall, measuring approximately two centimeters. Figure 1B is an axial view of the same computerized tomography scan, with arrows indicating the two-centimeter perforation and origin of the gastric leak with contrast.

**Figure 2:**
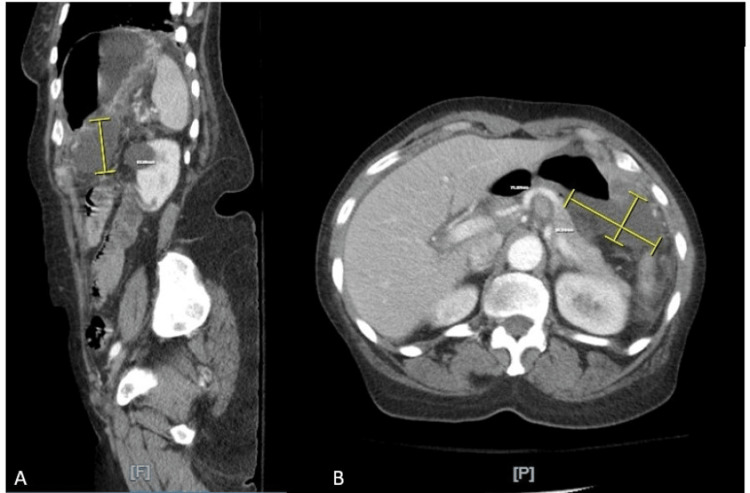
A computerized tomography scan of the abdomen and pelvis demonstrating a gastric leak in the lesser sac. Figure 2A is a sagittal view of a computerized tomography scan of the abdomen and pelvis with intravenous and oral contrast administration. There is leakage of gastric fluid and air into the lesser sac, measuring approximately five centimeters in height, as denoted in Figure 2A. Figure 2B is an axial view of the same computerized tomography scan in the delayed phase of contrast administration. There is enteric contrast leaking from the gastric wall posteriorly, measuring approximately seven centimeters long by four centimeters wide. The lesser sac fluid collection is, in total, 7 x 4 x 5 centimeters.

**Figure 3:**
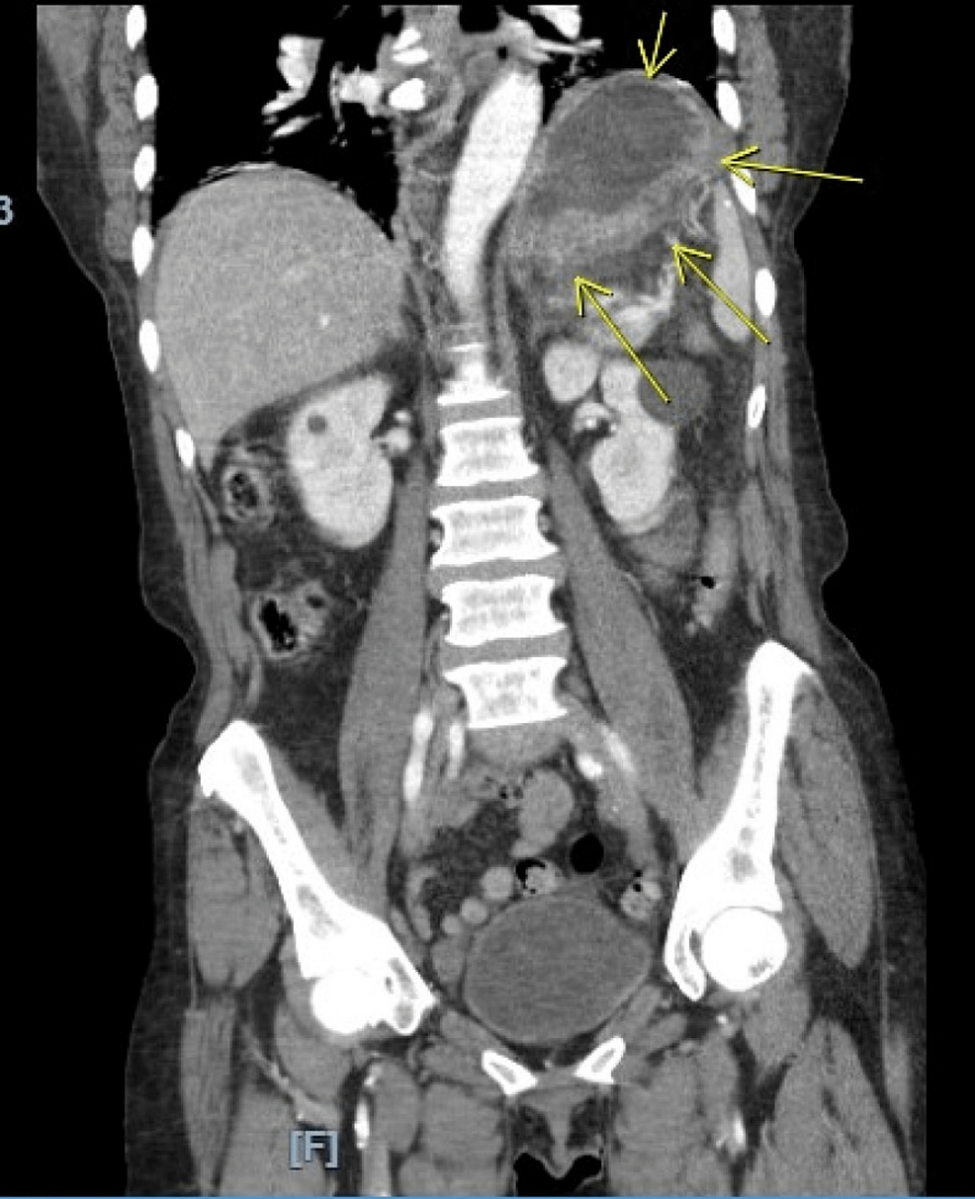
A computerized tomography scan of the abdomen and pelvis demonstrating a thickened gastric wall. A coronal view of the computerized tomography scan with oral and intravenous contrast administration is shown, with arrows indicating the highly vascularized and thickened gastric wall with mucosal hyperenhancement consistent with severe inflammation versus neoplastic involvement.

A comprehensive panel, lipase, and troponin were unremarkable. A computerized tomography (CT) scan of the abdomen and pelvis with intravenous contrast revealed a two-centimeter perforation along the posterior-inferior gastric wall (Figure 1), leakage of gastric fluid and air into the lesser sac (Figure 2), and severely thickened mucosal hyperenhancement of the gastric wall concerning for inflammation versus neoplastic involvement (Figure 3).

 

The patient was started on intravenous hydration and piperacillin-tazobactam, and surgery was consulted emergently. At this stage, there was concern that there was neoplastic involvement related to her CLL, although this presentation was atypical. She underwent a primary and Graham patch repair of the perforated gastric ulcer and an exploratory laparotomy with washout. A biopsy of the gastric ulcer revealed chronic active gastritis, positive for Helicobacter pylori (H. pylori), with dense lymphoplasmacytic infiltrates, small clonal kappa, CD5 positivity, with a B-cell population present in 8% of cells, and negative for CD10, BCL-6, and cyclin D1. Additionally, CD23 was not expressed, and kappa was moderately expressed. The phenotype of the T-cells (20% of the total) showed no pan-T-cell antigenic deletion. The CD4:CD8 ratio was inverted (0.4:1), and the CD13+/CD33+ granulocytes, monocytes, and histiocytes comprised 25.4%. This finding was consistent with minimal involvement by a CD5-positive B-cell lymphoproliferative disorder. Postoperatively, the patient developed hemolytic anemia with a drop in hemoglobin to 5.7 mg/dL, requiring transfusions and intravenous iron, and she was given stress-dose steroids with hydrocortisone 50 milligrams every 12 hours for two days. Ibrutinib was held due to the possible risk of neutropenia and delayed wound healing and/or bleeding. She was then transitioned to oral steroids, prednisone 40 milligrams daily, to be continued upon discharge.

She was discharged on antibiotics, omeprazole, and prednisone for her CLL. Ibrutinib was held for two weeks to allow for adequate wound healing. One month following discharge, the patient improved clinically and no longer had abdominal pain. She was restarted on ibrutinib. A repeat endoscopy was scheduled to biopsy and confirm H. pylori eradication.

## Discussion

Peptic ulcer disease (PUD) is typically caused by imbalances in acid homeostasis and mucosal defense barriers, with smoking, alcohol consumption, and steroid or non-steroidal anti-inflammatory drug (NSAID) use increasing the risk [4]. Only 2%-14% of peptic ulcers will perforate, and patients will present with acute abdominal pain, signs of peritonitis, and hemodynamic instability [4]. Early diagnosis with an erect chest radiograph and urgent surgical intervention is imperative [4]. Perforation of PUD confers high mortality, and recurrence of perforations is up to 12.2% [4]. The mortality of elderly patients (greater than 65 years of age) is 37.7% from a single perforation [4].

CLL causing a perforated PUD is rare [3]. Rare gastrointestinal complications often occur when CLL transforms into Richter syndrome, an aggressive diffuse large B-cell lymphoma that carries a poor prognosis [3]. Acute abdominal conditions, such as bleeding ulcers, esophagitis, intestinal perforations, or colitis, usually occur during relapsing episodes and more commonly in acute leukemias or Richter’s syndrome [2]. Chronic leukemias confer a higher risk of infiltration of lymphoreticular organs such as the spleen or liver [2]. Leukemic lesions of the ileum and proximal colon will be nodular, causing intussusception or intestinal obstruction, and ulcerative or plaques of the stomach causing bleeding [2]. Mantle cell lymphoma, marginal B-cell lymphoma, and follicular lymphoma have a higher incidence of colonic involvement [3]. Specifically, CLL may induce upper gastrointestinal bleeding through direct infiltration or via portal hypertensive varices [3]. Neutropenic enterocolitis (NE) is another major gastrointestinal complication of CLL therapy, in which chemotherapy may induce a necrotizing infection in the cecum, ascending colon, and terminal ileum [2]. Although our patient had confirmed PUD perforation, her symptoms mimicked those of NE, including fever, right lower quadrant pain, diarrhea, nausea, and vomiting [2]. Moreover, one of the treatments for CLL includes a tyrosine kinase inhibitor called ibrutinib [5, 6]. Although this medication has demonstrated high efficacy, good oral bioavailability, and high efficacy in those who are tyrosine kinase receptor naïve, one of the known side effects is bleeding [5, 6]. This can also contribute to upper gastrointestinal bleeding in patients with gastrointestinal involvement [5, 6]. Our patient was unique in that she had a perforated, rather than a bleeding peptic ulcer caused by infiltrative CLL. Given the biopsy findings, the perforated ulcer was caused by CLL rather than H. pylori; however, there may be an overlap in the inflammatory cascade causing chronic inflammation in H. pylori and CLL [7, 8].

## Conclusions

CLL affecting the gastrointestinal tract is uncommon, especially in the form of peptic ulcer perforation. In the upper gastrointestinal tract, bleeding predominates compared to the lower gastrointestinal tract, in which peritonitis or colitis can be seen. Our patient’s case was unique in that she had a perforated peptic ulcer in the setting of CLL infiltration after starting ibrutinib. This phenomenon is poorly understood, but future studies may warrant investigating the role of endoscopy for CLL surveillance and direct management in those with gastrointestinal involvement.

## References

[1] Rai KR, Jain P (2016). Chronic lymphocytic leukemia (CLL) - then and now. Am J Hematol.

[2] Ebert EC, Hagspiel KD (2012). Gastrointestinal manifestations of leukemia. J Gastroenterol Hepatol.

[3] Arkkila PE, Nuutinen H, Ebeling F, Elonen E, Kärkkäinen P, Karjalainen-Lindsberg ML (2008). Colonic involvement in a patient with chronic lymphocytic leukaemia. Gastroenterol Res Pract.

[4] Chung KT, Shelat VG (2017). Perforated peptic ulcer - an update. World J Gastrointest Surg.

[5] Zi F, Yu L, Shi Q, Tang A, Cheng J (2019). Ibrutinib in CLL/SLL: from bench to bedside (review). Oncol Rep.

[6] Kaur V, Swami A (2017). Ibrutinib in CLL: a focus on adverse events, resistance, and novel approaches beyond ibrutinib. Ann Hematol.

[7] Chen JP, Wu MS, Kuo SH, Liao F (2014). IL-22 negatively regulates Helicobacter pylori-induced CCL20 expression in gastric epithelial cells. PLoS One.

[8] Delchier JC, Lamarque D, Levy M (2001). Helicobacter pylori and gastric lymphoma: high seroprevalence of CagA in diffuse large B-cell lymphoma but not in low-grade lymphoma of mucosa-associated lymphoid tissue type. Am J Gastroenterol.

